# Substituted Two- to Five-Ring Polycyclic Aromatic Compounds Are Potent Agonists of Atlantic Cod (*Gadus morhua*) Aryl Hydrocarbon Receptors Ahr1a and Ahr2a

**DOI:** 10.1021/acs.est.1c02946

**Published:** 2021-11-05

**Authors:** Roger Lille-Langøy, Kåre Bredeli Jørgensen, Anders Goksøyr, Daniela M. Pampanin, Magne O. Sydnes, Odd André Karlsen

**Affiliations:** † Department of Biological Sciences, University of Bergen, N-5020 Bergen, Norway; ‡ Department of Chemistry, Bioscience and Environmental Engineering, University of Stavanger, N-4036 Stavanger, Norway

**Keywords:** alkylated PAH, methylchrysene, chrysenol, PAH metabolites, reporter gene assay

## Abstract

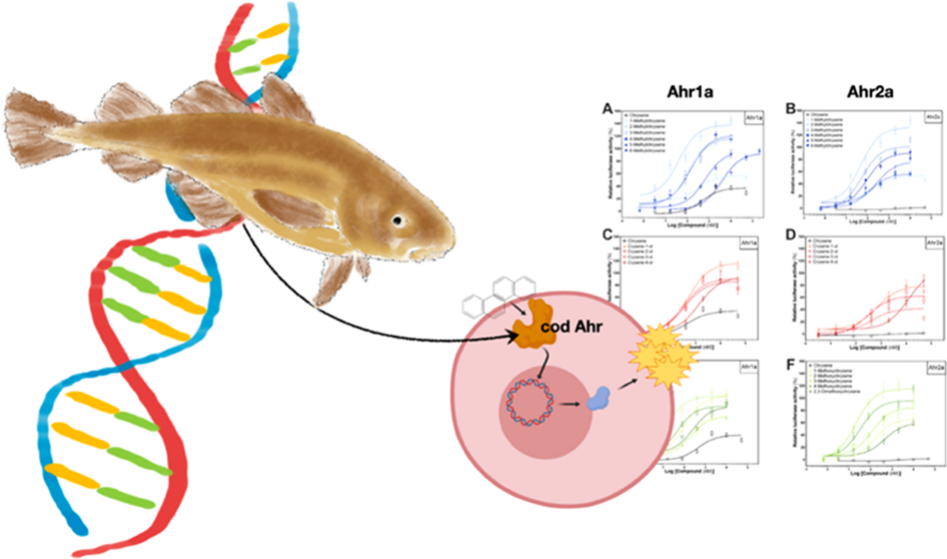

Polycyclic aromatic hydrocarbons (PAHs) are among the most toxic and bioavailable components found in petroleum and represent a high risk to aquatic organisms. The aryl hydrocarbon receptor (Ahr) is a ligand-activated transcription factor that mediates the toxicity of 2,3,7,8-tetrachlorodibenzo-*p*-dioxin (TCDD) and other planar aromatic hydrocarbons, including certain PAHs. Ahr acts as a xenosensor and modulates the transcription of biotransformation genes in vertebrates, such as cytochrome P450 1A (*cyp1a*). Atlantic cod (*Gadus morhua*) possesses two Ahr proteins, Ahr1a and Ahr2a, which diverge in their primary structure, tissue-specific expression, ligand affinities, and transactivation profiles. Here, a luciferase reporter gene assay was used to assess the sensitivity of the Atlantic cod Ahrs to 31 polycyclic aromatic compounds (PACs), including two- to five-ring native PAHs, a sulfur-containing heterocyclic PAC, as well as several methylated, methoxylated, and hydroxylated congeners. Notably, most parent compounds, including naphthalene, phenanthrene, and partly, chrysene, did not act as agonists for the Ahrs, while hydroxylated and/or alkylated versions of these PAHs were potent agonists. Importantly, the greater potencies of substituted PAH derivatives and their ubiquitous occurrence in nature emphasize that more knowledge on the toxicity of these environmentally and toxicologically relevant compounds is imperative.

## Abstract 2

Substituted PAH congeners act as strong aryl hydrocarbon receptor agonists, emphasizing the need to consider these compounds in environmental monitoring and risk assessment of aquatic environments.

## Introduction

Polycyclic aromatic compounds (PACs) are a diverse group of chemicals that contain aromatic rings organized in linear, angular, or clustered structures. PACs include polycyclic aromatic hydrocarbons (PAHs) and also nitrogen-, oxygen-, or sulfur-containing heterocyclic aromatic compounds (NSO-PACs), as well as compounds with heteroatoms containing functional groups (such as quinones, nitro-PAHs, and hydroxy-PAHs).^1−3^ PAHs originating from fossil fuels (petrogenic) and incomplete combustion of organic matter (pyrogenic) are frequently present in aquatic environments.^4^ Petrogenic PAHs often originate from manmade sources like discharges of industrial and urban effluents, shipping, offshore oil drilling, oil refineries, and accidental oil spills.^5,6^ Historically, pyrogenic PAHs have originated from wood treatment facilities, where creosote was used.^7^

In general, PACs have low water solubility and are mainly found associated with suspended particulate matter in water and do eventually accumulate in sediments.^4^ However, some PAHs (with *K*_ow_ > 6) tend to bioaccumulate in fish. Nevertheless, these PAHs have relatively short half-lives due to efficient metabolism and excretion and thus do not biomagnify.^8^ In vertebrates, such as fish, birds, and mammals, hepatic cytochrome P450 monooxygenase enzyme systems are mostly responsible for this rapid metabolism. Due to the carcinogenic and mutagenic properties of PAH metabolites, PAHs can cause adverse effects in aquatic organisms and potentially to humans through fish and shellfish consumption.^9−15^ For these reasons, they have been regarded as high priority compounds for environmental pollution monitoring, and a priority list of 16 PAHs (PAH-16) was made by the US Environmental Protection Agency (EPA).^16^ Today, PAH-16 are routinely analyzed in environmental monitoring programs and risk assessments of PAH-polluted sites.

In addition to the unsubstituted parent compounds, substituted PAHs, such as alkylated PAH derivatives, can be found in the environment, and these have been reported to be more toxic than their unsubstituted congeners.^5,17^ Substituted PAHs have been shown to contribute to the toxicity of both pyrolytic and petrolytic PAH mixtures in the early life stages of rainbow trout (*Oncorhynchus mykiss*), Japanese medaka (*Oryzias latipes*), and zebrafish (*Danio rerio*).^18−22^ Hydroxylation of alkylated phenanthrenes has also been shown to enhance early life stage toxicity in Japanese medaka,^23^ further emphasizing the importance of considering the contribution of substituted PAHs in mediating toxicity in fishes and other aquatic organisms. Furthermore, as most PAHs are subject to metabolic activation by cytochrome P450s and epoxide hydrolases, epoxide and diol metabolites are formed *in vivo* in vertebrates.^5,24,25^ The major PAH oxidation products formed in fish are *trans*-dihydrodiols, including (1*R*,2*R*)-1,2-dihydrochrysene-1,2-diol, (1*R*,2*R*)-1,2-dihydrophenantrene-1,2-diol, and (1*R*,2*R*)-1,2-dihydronaphthalene-1,2-diol, which are some of the *trans*-dihydrodiols derived from chrysene, phenanthrene, and naphthalene, respectively.^11,26,27^ In addition to being formed during biotransformation, hydroxylated PAHs may also be produced during incomplete combustion of, *e.g*., fire wood.^28^ Notably, the toxic potential of alkylated and oxygenated PACs has received less attention compared to the 16 PAHs prioritized for environmental monitoring.^1^ As the PAH-16 only encompass parent PAHs, the importance of expanding our knowledge of the toxicities of heterocycles and alkyl derivatives and include such compounds in a more extensive panel of PACs for environmental monitoring has been proposed.^29^

The toxicity of PACs has, to a large extent, been attributed to the activation of the aryl hydrocarbon receptor (AHR) and the subsequent alteration of its target gene expression.^30−33^ AHR is a ligand-activated transcription factor and a member of the basic helix–loop–helix PER-ARNT-SIM (bHLH-PAS) superfamily, which has been widely studied because of its important role in mediating cellular responses to halogenated aromatic hydrocarbons. 2,3,7,8-tetrachlorodibenzo-*p*-dioxin (TCDD) has been established as the most potent exogenous agonist for various AHR orthologs. AHR-dependent toxicities are both species- and tissue-specific and can cause a wide spectrum of effects, including teratogenicity, immuno-, hepato-, cardio- and dermal toxicity, modulation of cell growth, proliferation and differentiation, endocrine disruption, and tumor promotion.^33^ Accordingly, several adverse outcome pathways involving AHR and/or activation of AHR as the molecular initiating event have been described and are currently being developed (e.g., AOP21 and AOP150, https://aopwiki.org/aops/). To abide to different nomenclature rules for mammalian and fish proteins,^34,35^ abbreviated protein names have two different formats in scientific publications. Thus, AHR or Ahr is used when referring to mammalian or fish aryl hydrocarbon receptor orthologs, respectively. Ligand-activated AHR heterodimerizes with the aryl hydrocarbon receptor nuclear translocator (ARNT) and specifically binds to xenobiotic response elements (XREs) upstream of the AHR target genes, modulating the transcription of a battery of genes encoding enzymes involved in the biotransformation of xenobiotics, including CYP1A.

Atlantic cod is a culturally, ecologically, and economically important teleost species residing in the Barents Sea, the North Atlantic Ocean, and the Baltic Sea.^36^ Recently, two Ahr proteins were identified and functionally characterized in Atlantic cod, *i.e.*, Ahr1a and Ahr2a. The Ahr paralogs differ in both tissue-specific and spatiotemporal gene expression, ligand-binding affinities, and transactivation activities, suggesting that Ahr1a and Ahr2a have acquired different functional roles in Atlantic cod through a process of subfunction partitioning.^37,38^ Moreover, current data suggests that Ahr2a is the main subtype involved in mediating responses to xenobiotics, while Ahr1a appears to be important in the development of the eye in cod embryos and larvae. However, the high sensitivity of Ahr1a to different ligands, including benzo[*a*]pyrene, suggests that Ahr1a activity can be modulated by pollutants.

As the AHR/Ahr pathway plays a central role in PAH-mediated toxicity, it is necessary to obtain knowledge of the sensitivities of Atlantic cod Ahrs to the wide array of PACs present in the environment. In this study, a total of 31 compounds were tested for their ability to transactivate the Atlantic cod Ahr1a and Ahr2a *in vitro*, and their sensitivities and efficacies were compared. Among the 31 PACs, seven were unsubstituted and represented congeners previously detected in Atlantic cod liver and bile, as well as PACs that are major constituents of crude oil, such as naphthalene, phenanthrene, fluorene, and chrysene.^5,39^ Moreover, substituted versions of chrysene and phenanthrene are abundant constituents of petrogenic substances present in marine environments, and an extensive library of alkylated phenanthrenes and substituted chrysenes, including dimethylated phenanthrenes as well as methylated, methoxylated, and hydroxylated chrysene congeners, has therefore been assessed in this study. Finally, *trans*-diols of naphthalene and phenanthrene, which are considered the most prominent biotransformation products of these compounds commonly found in fish bile, are included in these analyses, and to our knowledge, this is the first time these metabolites have been evaluated as agonists for Ahr.^40^ Importantly, although *in vitro* Ahr activation not necessarily correlates to adverse outcomes in organisms, reporter gene assays as applied in this study may still expand our understanding of PAC-mediated toxicities. Such data could also prove important for future risk assessment and further reveal functional differences between receptors and receptor subtypes and potentially divergences in species susceptibility to PAC exposure.^5,39^

## Materials and Methods

### Chemicals

2,3,7,8-tetrachlorodibenzo-*p*-dioxin (TCDD) was purchased from LGC Standards (Teddington, U.K.). The PAHs chrysene (CHR), 5-methylchrysene (5-MC), phenanthrene (PHE), naphthalene (NAP), pyrene (PYR), benzo[*a*]pyrene (BAP), fluorene (FLU), (1*R*,2*R*)-1,2-dihydronaphthalene-1,2-diol (*R*,*R*-1,2-DHN), and dibenzothiophene (DBT) were purchased from Merck KGaA (Darmstadt, Germany). The synthesis of (1*R*,2*R*)-1,2-dihydrophenanthrene-1,2-diol (*R*,*R*-1,2-DHP),^11^ 1-,2-,3-,4-methoxychrysene (1-,2-,3-,4-MOC) and chrysene-1-*ol*,-2-*ol*,-3-*ol*,-4-*ol* (1-,2-,3-,4-COH),^41^ 1-,2-,3-,4-,6-methylchrysene (1-,2-,3-,4-,6-MC),^42^ 3-ethylphenanthrene (3-EP), and 3-propylphenanthrene (3-PP)^43^ is described elsewhere. 1,7-, 2,3-, and 2,7-dimethylphenanthrene (1,7-, 2,3-, and 2,7-DMP) were prepared as described by Böhme et al.,^44^ and the preparation of 2,3-dimethoxychrysene (2,3-DMOC) is described in the Supporting Information.

### Transfection, Exposure, Luciferase Reporter Gene Assay, and Viability Assay

COS-7 simian kidney cells were seeded onto 96 well plates (10 000 cells/well) in Dulbecco’s modified Eagle’s medium (DMEM) with phenol red, supplemented with 10% fetal bovine serum (FBS), 4 mM l-glutamate, 1 mM sodium pyruvate, and 100 U/mL penicillin–streptomycin (Merck KGaA, Darmstadt, Germany), and cultivated at 37 °C with 5% CO_2_ for 24 h. Cells were transiently co-transfected with a eukaryotic expression plasmid (pcDNA3.1/Zeo(+), 31 ng/well), pcDNA3.1/Zeo(+)-based plasmids encoding gmAhr1a or gmAhr2a (3 ng/well), gmArnt1 (6 ng/well),^37^ a luciferase reporter plasmid containing four DREs (pGudLuc6.1, 30 ng/well), and a β-galactosidase normalization plasmid (pCMV-βGAL, 30 ng/well),^45,46^ using a Mirus TransIT LT-1 transfection reagent according to the recommendations of the supplier. COS-7 cells were seeded and cultivated in DMEM (4500 mg/L glucose), supplemented with 10% FBS, 1 mM sodium pyruvate, and 4 mM l-glutamine. In the exposure medium, FBS was substituted with 10% charcoal-stripped FBS (VWR International, Radnor). To create the exposure media with the highest test concentration, compounds solved in dimethyl sulfoxide (DMSO) were diluted 1:200 in exposure medium, resulting in exposure media with 1× test compound and 0.5% DMSO. The highest concentration exposure media were serially diluted five times (1:2 for the first dilution, then 1:5) in exposure medium supplemented with 0.5% DMSO. Following transfection, cells were incubated in exposure media containing PACs or solvent control (0.5% DMSO) for 24 h. Reporter gene assays were repeated at least three times and with three technical replicates per exposure. TCDD (30 pM to 100 nM) was used as a known agonist in each experiment. Absorbance and luminescence measurements were performed on an EnSpire Multimode plate reader (PerkinElmer, Waltham, MA). While no wavelength filtering was used to measure firefly luciferase activity by luminescence, absorbance measurements to quantify β-galactosidase activity were performed at 420 nm wavelength. The viability of exposed cells was evaluated using the resazurin reduction assay as previously described.^47,48^

### Data Analysis and Statistics

Recorded luminescence was normalized for variation in transfection efficiencies using β-galactosidase enzyme activity. The difference in normalized light units measured in lysates from exposed cells to solvent-exposed cells was calculated and expressed relative to the maximum response in TCDD-exposed cells. Response curves were prepared by nonlinear regression analyses in Prism v7 and used for the determination of half-maximal effective concentration values (EC_50_) and maximum efficacies (*E*_max_). EC_50_ values were only determined for compounds that produced a sigmoidal concentration–response curve. Relative effect potencies 25 (REP_25_) were determined by dividing the EC_25_ of TCDD by the concentration of PAC necessary to produce a response equal to 25% of the response of TCDD with gmAhrs, essentially as described by Villeneuve et al. and Lam et al.^49,50^ The D’Agostino–Pearson (Ahr activation data) or Kolmogorov–Smirnov (viability data) normality tests were used to confirm the normal distribution of the data. One-way analysis of variance (ANOVA) and Dunnett′s test were used to compare responses at different concentrations of compounds and solvent control mediated via the same Ahr, in addition to comparing metabolic activity in PAC-exposed cells and cells exposed to solvent control in the resazurin reduction assay. Welch′s t-test was used to compare the maximum Ahr-mediated response and potency (*E*_max_ and EC_50_) between Ahr1a or Ahr2a produced by the same test compound (Prism v7).

## Results

### *In Vitro* Transactivation of Atlantic Cod Ahrs

Luciferase reporter gene assays were used to assess the ability of 31 PACs to transactivate the Atlantic cod Ahr1a and Ahr2a *in vitro*. The test panel consisted of seven unsubstituted two- to five-ring PACs, including NAP, FLU, PHE, CHR, PYR, BAP, and the heterocyclic sulfur-containing dibenzothiophene, as well as 24 methylated, hydroxylated, and methoxylated derivatives of chrysene, phenanthrene, and naphthalene (Table 1). The transactivation profiles of the unsubstituted and substituted PAHs were calculated relative to the *E*_max_ determined for TCDD (Suppporting Figure S1).

**Table 1:**
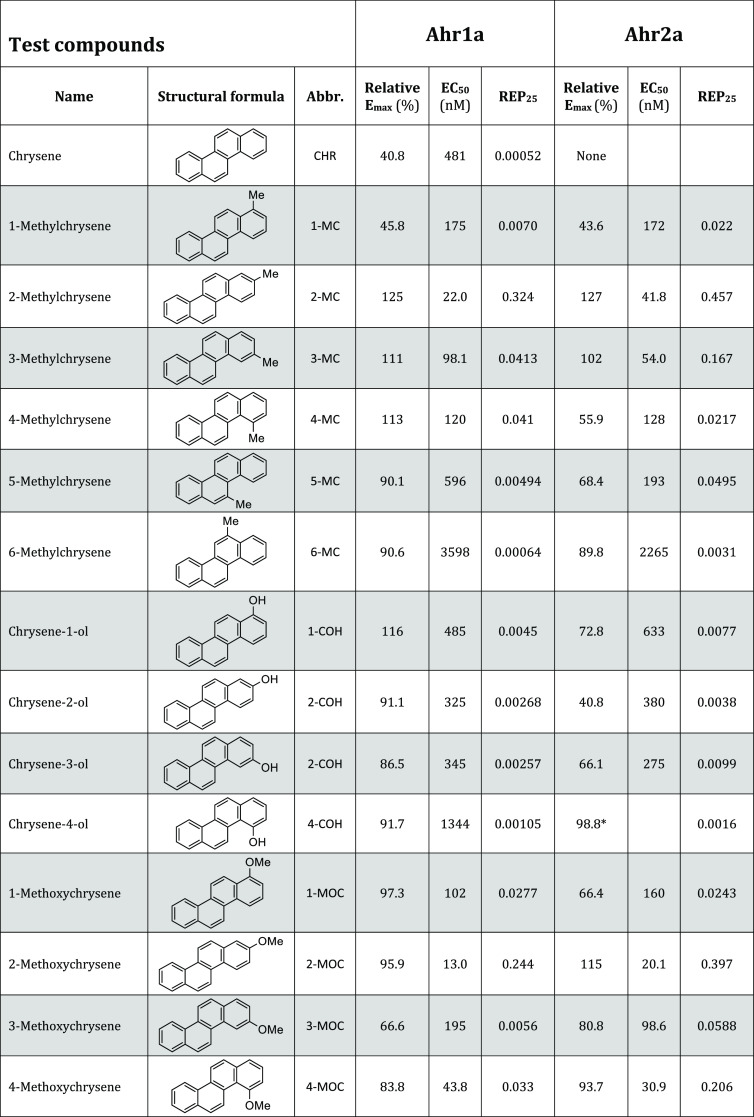

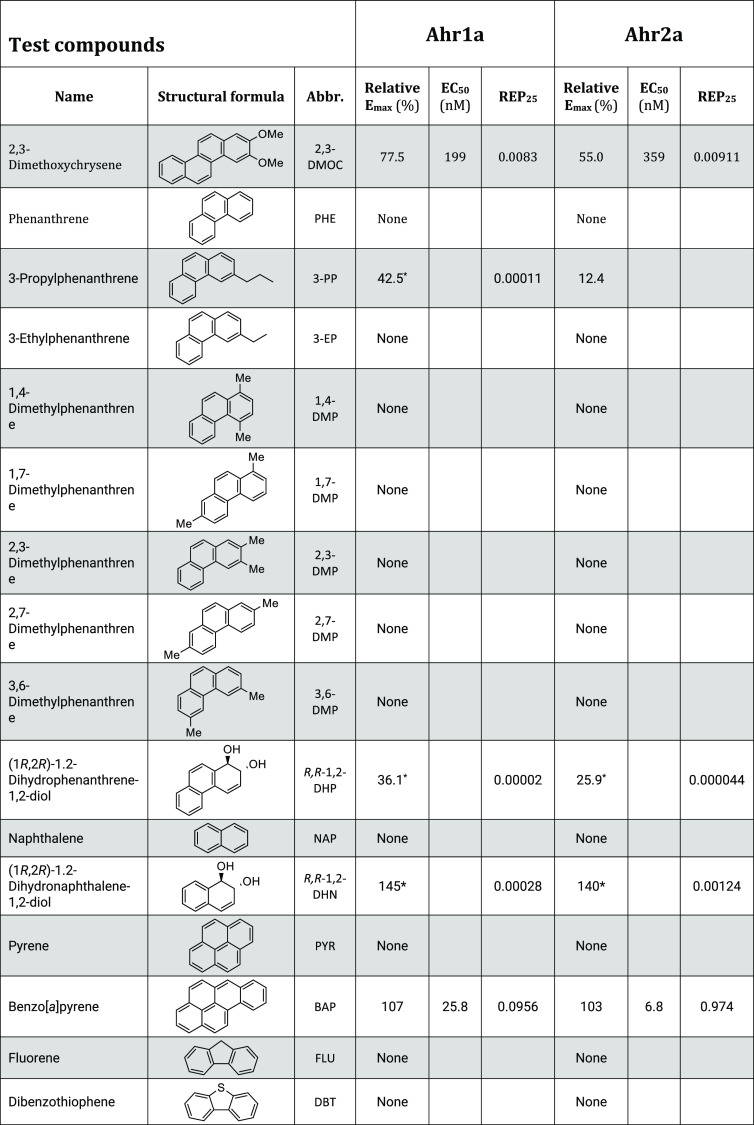

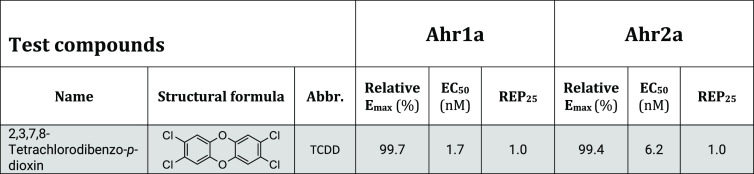
*In Vitro* Transactivation of Atlantic Cod Aryl Hydrocarbon Receptors (Ahrs), Ahr1a and Ahr2a, in a Luciferase-Based Ligand Activation Assay^a^

a Cells expressing either Ahr1a or Ahr2a were exposed to single polycyclic aromatic compounds and a control agonist (TCDD). Response curves were fitted by nonlinear regression using GraphPad Prism 7.0. Efficacies produced by the tested compounds are expressed in percent of the maximum efficacy determined for TCDD (relative *E*_max_), while potencies were calculated as half-maximal effective concentration (EC_50_) and relative potencies 25 (REP_25_). For response curves that did not reach a plateau for the range of selected concentrations, the efficacy was given as the relative response at the highest tested concentration, and for these responses, the EC_50_ was not calculated (indicated with *). Exposures that did not produce agonistic responses significantly different from solvent-exposed cells have been denoted “None”.

**Figure 1:**
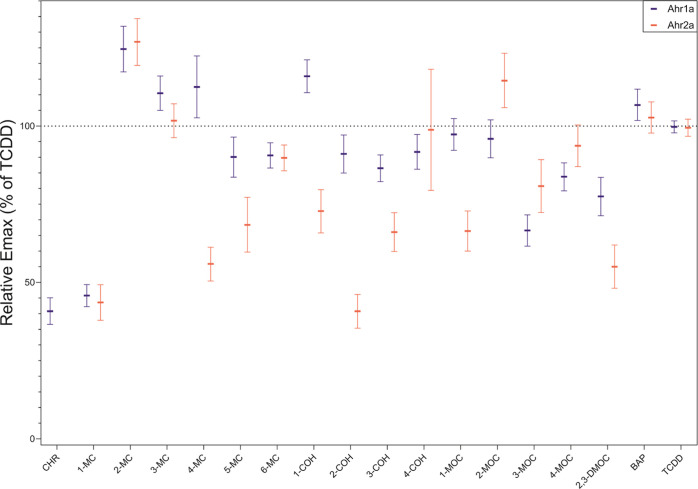
Calculated efficacies for the *in vitro* transactivation of Atlantic cod aryl hydrocarbon receptors (Ahr1a (blue) and Ahr2a (red)) by PACs as indicated. Responses produced by PACs were compared to the maximum effect (*E*_max_) mediated by TCDD. *E*_max_ was determined from data originating from three or more individual experiments with three technical replicates using three-parameter nonlinear regression (GraphPad Prism, v7.0) and is presented as means with 95% confidence intervals.

**Figure 2:**
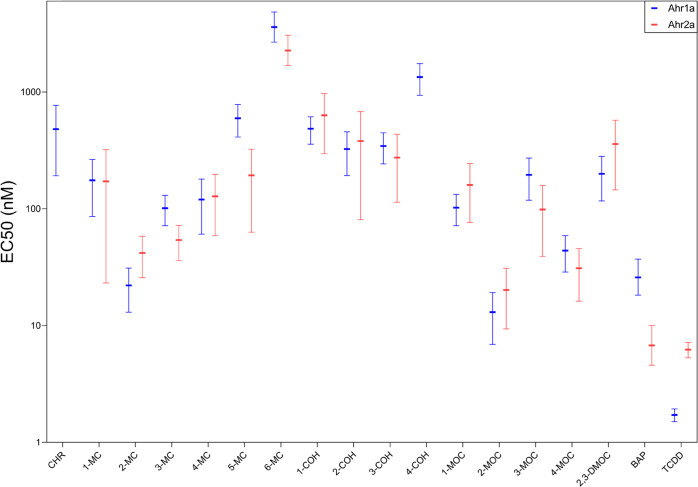
Calculated potencies for the *in vitro* transactivation of Atlantic cod aryl hydrocarbon receptors (Ahr1a (blue) and Ahr2a (red)) by PACs as indicated. Half-maximal effective concentration 50 (EC_50_) was determined from data from three or more individual experiments with three technical replicates using three-parameter nonlinear regression (GraphPad Prism, v7.0) and is presented as mean with corresponding 95% confidence intervals.

**Figure 3:**
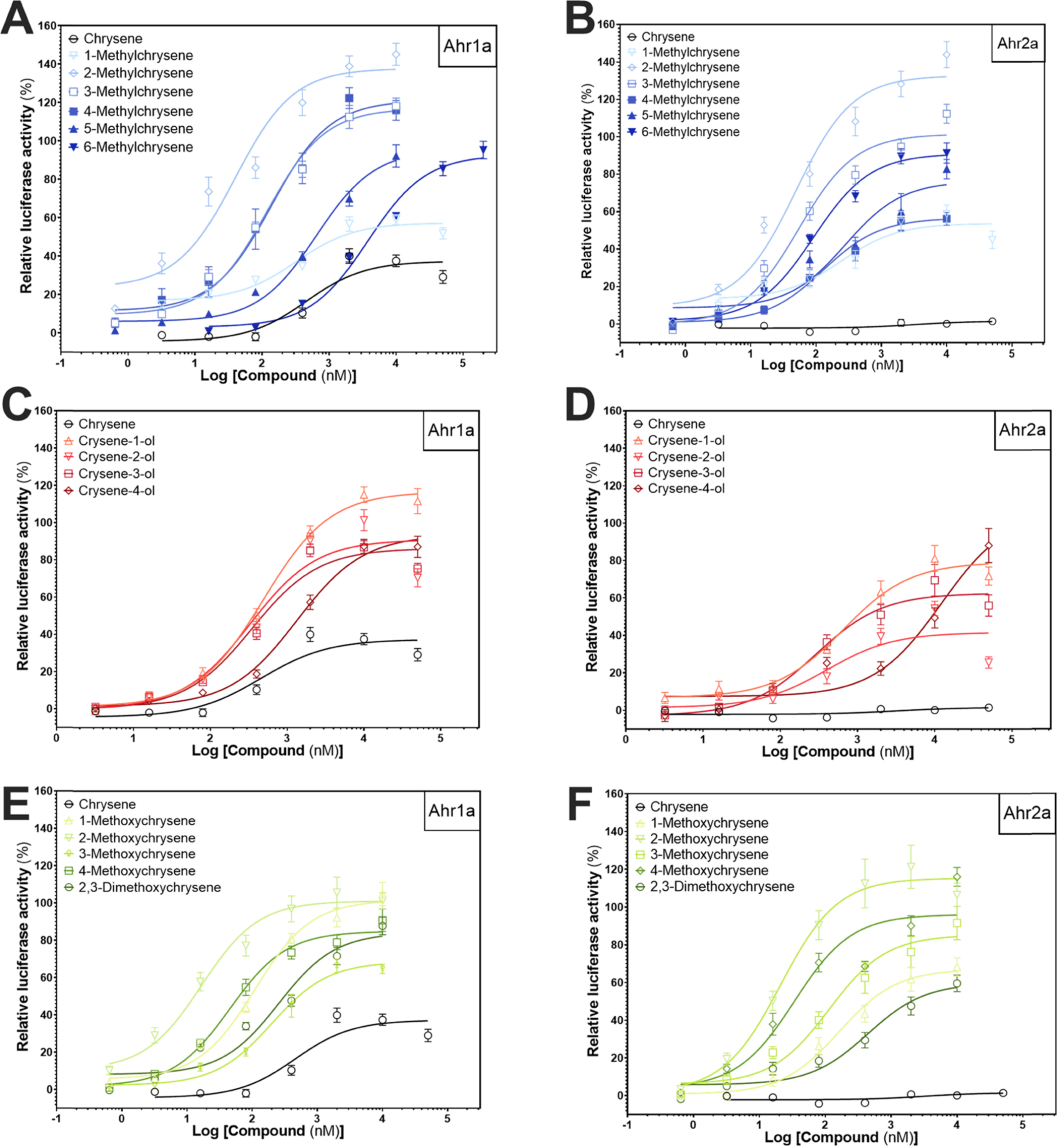
*In vitro* transactivation of Atlantic cod aryl hydrocarbon receptors (Ahrs) by unsubstituted and substituted chrysenes. Response curves were recorded with luciferase-based Ahr1a and Ahr2a ligand activation assays for unsubstituted chrysene, methyl- (A, B) hydroxy- (C, D), and methoxychrysenes (E, F). Responses are expressed relative to the maximum response induced by TCDD (equals 100%).

Twenty-one and 20 PACs were found to activate Ahr1a and Ahr2a, respectively (Supporting Tables S1–S5). Eleven PACs (NAP, FLU, PYR, DBT, PHE, 3-EP, 1,4-, 1,7-, 2,3- 2,7-, and 3,6-DMP) did not activate either Ahr1a or Ahr2a. Receptor efficacies (*E*_max_) and potencies (EC_50_ and REP_25_) are visualized in Figures 1 and 2 and summarized in Table 1. Notably, BAP and CHR were the only unsubstituted compounds that acted as Ahr agonists. In accordance with previous data, BAP was an agonist for both Ahrs,^37^ while chrysene was shown here to solely activate Ahr1a (Supporting Figure S2 and Figure 3). Intriguingly, most of the substituted PACs activated the two Ahr proteins. Moreover, the hydroxylated or propylated two- and three-ring PACs, including *R*,*R*-1,2-DHN, *R*,*R*-1,2-DHP, and 3-PP, activated both Ahr1a and Ahr2a, in contrast to their parent compounds (Supporting Figure S3). Furthermore, six monomethylated chrysenes, four chrysenols, four methoxychrysenes, and 2,3-DMOC also activated the two Ahrs (Figure 3A–F). The substituted chrysenes demonstrated, in general, higher efficacy and potency in comparison to chrysene-mediated Ahr1a activation (Figures 1 and 2, Supporting Tables S2–S4).

BAP and three substituted PACs (*i.e.*, 2-MC, 4-MC, and 1-COH) produced higher efficacies (superefficacies) than TCDD with Ahr1a (Table 1, Supporting Figure S2, Supporting Table S6), while 3-MC, 5-MC, 6-MC, 2-COH, 4-COH, 1-MOC, and 2-MOC were agonists with efficacies similar to that of TCDD. In contrast, CHR, 1-MC, 3-COH, 3-MOC, 4-MOC, and 2,3-DMOC produced lower efficacies than TCDD via Ahr1a (Supporting Table S6). Similarly, for responses via Ahr2a, two of the tested compounds (2-MC and 2-MOC) elicited superefficacies that were 127 and 115% of the TCDD maximum induction, while five of the tested compounds (3-MC, 6-MC, 4-COH, 4-MOC, and BAP) produced similar efficacies (Table 1, Supporting Table S6). Ten PACs (CHR, 1-MC, 4-MC, 5-MC, 1-COH, 2-COH, 3-COH, 1-MOC, 3-MOC, and 2,3-DMOC) demonstrated lower efficacies in comparison to TCDD (Table 1, Supporting Table S6). REP_25_ values were calculated to express relative potencies of the PACs to TCDD with the two cod Ahrs, and as expected, none of the PACs were more potent agonists than TCDD for either Ahr1a or Ahr2a (Table 1). However, BAP approached TCDD with a REP_25_ of 0.97 (TCDD = 1.0) with Ahr2a. With this subtype, five compounds (2-MC, 3-MC, 2-MOC, 4-MOC, and BAP) demonstrated REP_25_ values of 0.17–0.97, whereas with Ahr1a, only 2-MC and 2-MOC were in the same range (Table 1). In general, REP_25_ values were comparable among the two receptors, with the exception of 5-MC, 6-MC, 3-MOC, 4-MOC, *R,R*-1,2-DHN, and BAP, which all displayed a 5- to 10-fold higher REP_25_ with Ahr2a compared to Ahr1a.

The resazurin reduction assay was used to monitor potential effects on the metabolic activities in COS-7 cells after PAC exposure. While the majority of the compounds did not affect cell viability, a slight reduction in metabolic activities in cells exposed to the highest concentrations used of 5-MC, 6-MC, 1-COH, 3-COD, 3-MOC, 4-MOC, and 2,3-DMP was observed (Supporting Figure S4).

## Discussion

The cloning of Atlantic cod Ahr1a and Ahr2a was recently described, along with the activation of these receptors by several well-known mammalian and teleost AHR/Ahr agonists, including TCDD, β-naphthoflavone, BAP, FICZ, and PCB-126.^37^ In the current study, a selection of 31 PACs, consisting of 6 unsubstituted, 24 substituted, and 1 heterocyclic PAC, was assessed for their ability to activate cod Ahra1a and Ahr2a, and their efficacies and potencies were compared. The recorded transactivation data revealed that most of the PACs activated the two Ahrs and that the list of compounds that activated Ahr1a or Ahr2a was largely overlapping. The only exception was unsubstituted chrysene, which only activated Ahr1a. Most importantly, monomethylation, monohydroxylation, and mono- and dimethoxylation of chrysenes, as well as hydroxylation of naphthalenes and phenanthrenes, appear to significantly increase their agonistic potential. In several cases, including NAP and PHE, the unsubstituted congener did not act as an Ahr agonist, which is in agreement with previous data reporting that two- and three-ring unsubstituted PAHs are generally inactive in fish, avian, and mammalian systems.^40,51^

Environmental exposures to PAHs most often involve mixtures rather than single compounds, and dependent on their sources, the content of substituted and unsubstituted PAHs varies to a large extent.^5^ While pyrogenic PAHs usually are unsubstituted, petrogenic PAHs are largely alkyl-substituted and parental PAHs only comprise a minor fraction of such mixtures. Phenanthrene and its derivatives are major components in crude oil and occur in sediments at high concentrations.^52,53^ Both phenanthrene and its alkyl derivatives, such as retene, have been shown to affect early life stages of fish, while chronic exposures to these compounds have resulted in deformities, edemas, and embryo mortality in zebrafish.^54,55^ While phenanthrene is considered a poor AHR/Ahr agonist and phenanthrene-mediated toxicities in early life stages of fish are assumed to be Ahr independent,^56−59^ it has been demonstrated *in vitro* that mono- and dimethylated phenanthrene are more potent agonists of rat and human AHR than phenanthrene.^60,61^ Although examined in a limited number of teleost species, monomethylated phenanthrenes, including 1-methylphenanthrene and 4-methylphenanthrene, appear not to be able to efficiently activate Ahr or induce Cyp1a activity in fish.^62,63^ Similarly, none of the five dimethylated phenanthrenes assessed in this study were able to activate the two Ahrs, including 3,6-DMP that previously was shown to activate human AHR.^60^ The alkylated phenanthrene structures, 2-ethylphenanthrene and 9-ethylphenanthrene, have previously been characterized as weak inducers of ethoxyresorufin-O-dealkylase (EROD) activity in waterborne exposures of juvenile rainbow trout.^64^ However, the related 3-ethylphenanthrene compound was not able to transactivate the Ahrs in the current study. Notably, 3-PP transactivated both Ahr1a and Ahr2a as a weak agonist, supporting that some alkylated phenanthrenes can activate the Ahr-signaling pathway in fish.

CHR and methylchrysenes originate from both pyrogenic and petrogenic sources, and methylchrysenes can also be formed from chrysene by bioalkylation.^65,66^ CHR has previously been found in mollusks, crustaceans, and fish and appears to be diluted in the marine food webs.^67−69^ CHR and its derivatives have been shown to activate rat and human AHR *in vitro*(70−73) and to induce Cyp1a activity in desert topminnow (*Poeciliopsis lucida*) hepatoma cells,^49^ and *in vivo* studies have indicated that CHR can activate both Ahr1 and Ahr2 in zebrafish.^74^ Unsubstituted CHR, in the current study, was found to act solely as an agonist of Ahr1a. However, each of the assessed monomethylated chrysenes was found to activate both Ahr1a and Ahr2a. Furthermore, all of the monomethylated chrysenes that were assessed produced increased efficacies in comparison to unsubstituted CHR. The high activities of the methylated chrysenes are in line with previous studies, where 1-MC and 5-MC have been shown to contribute significantly to the total TEQ value of PAH-contaminated environmental samples.^66^ When expressing the toxic potential of CHR and its derivatives relative to the toxicity of BAP (toxic equivalent factor; TEF), Richter-Brockmann and Achten noted that 1-MC and 5-MC had an agonistic potential of 10 and 100 times higher than CHR (TEF_Chr_ = 0.01, TEF_1-MC_ = 0.1, TEF_5-MC_ = 1.0).^66^ While our data showed that the six methylchrysenes were moderate to strong agonists of the Ahrs, we did not observe an evident correlation between efficacies and potencies of chrysene and methylated chrysenes and the previously reported TEF values for these compounds. However, these discrepancies may be ascribed to species-specific differences in ligand recognition and binding affinities between Atlantic cod Ahrs and human AHR. 1–6 MCs have also previously been demonstrated to activate rat AHR in the H4IIE-*luc* reporter gene assay, and REP_25_ values were calculated for these compounds.^50,73^ As REP_x_ values are based on internal relative comparisons of potency (EC_x_ values) to that of a reference compound (here, TCDD) with an assay-specific receptor, comparing REP values across studies and receptors is problematic. Both assay conditions, reporter system, and the sensitivity of the species studied will influence the baseline EC_50_. The EC_50_ for rat AHR with TCDD in the H4IIE-*luc* reporter assay is, in most cases, reported to be in the 8–18 pM range,^75,76^ whereas in our assay, we determined a TCDD EC_50_ of 1.7 and 6.2 nM with cod Ahr1a and Ahr2a, respectively, in the same range, as reported by Aranguren-Abadía et al.^37^ Taking this into account, REPs are still useful in comparing the relative transactivating potency of compounds across studies, species, and receptors. It is, for example, interesting to note that with the 1–6 MC series, Machala et al. found 3-MC to give the highest REP values, followed by 6-MC, 4-MC, 2-MC, 5-MC, and 1-MC. In our study, with cod Ahr2a, we found 2-MC to be the most potent, followed by 3-MC, 5-MC, 1-MC, 4-MC, and 6-MC. The REPs observed for MCs with cod Ahr2a also covered a larger range (150-fold) compared to that for rat AHR (40-fold). These differences in REPs between compounds may point to interesting differences in the structural features of the ligand-binding pocket of cod Ahrs versus rat AHR.

Furthermore, we also demonstrated that intermediates formed in the synthesis of chrysenols, such as mono- and dimethoxylated chrysene, produced high efficacies and were potent Atlantic cod Ahr agonists. This is also in accordance with a previous report, where 2-MOC has been shown to be a stronger agonist of rat AHR than chrysene.^50^

Fish have a high capacity to metabolize PAHs,^77^ and PAH-mediated activation of Ahr induces the expression of phase 1 and phase 2 biotransformation enzymes important for their elimination.^78^*trans*-dihydrodiol metabolites formed by CYP-mediated oxygenation are major hepatic oxidation products of PAHs and are excreted in the bile of bony fishes.^40,79,80^ The most abundant *trans*-dihydrodiols identified in Atlantic cod exposed to crude oil are *R*,*R*-1,2-DHP and *R*,*R*-1,2-DHN that are derived from phenanthrene and naphthalene, respectively.^11,26^ Noteworthy, we found that the *trans*-dihydrodiols of naphthalene and phenanthrene can act as agonists for the Ahrs, which was in contrast to their parent compounds that did not activate either Ahr1a or Ahr2a. To our knowledge, this is the first time that the biotransformation products of two- and three-ring compounds have been shown to act as Ahr agonists in a vertebrate organism. This observation emphasizes the promiscuity of the Atlantic cod Ahr ligand-binding pockets, which accommodate the recognition and binding of PAHs ranging from two- to at least five-ring structures. Intriguingly, activation of the Ahr-signaling pathway by the nonactive naphthalene and phenanthrene may thus occur after *in vivo* exposure, while their Ahr-activating properties should probably be ascribed to the *trans*-dihydrodiol metabolites formed after CYP-mediated hydroxylation of the two mother compounds.

Chrysenols, such as 1-COH, 4-COH, and 6-COH, are other examples of PAH metabolites that have been detected in fish. Chrysene-1-ol was detected in the bile of juvenile turbot (*Scophthalmus maximus*) that were exposed to various PAH mixtures,^77^ while 4-COH and 6-COH were found to constitute 6–9% of the metabolites detected in liver microsomes prepared from rainbow trout exposed to CHR.^81^ Notably, it has been reported that the exposure of zebrafish embryos to 2-COH and 6-COH caused circulatory, cardiac, and ocular effects, in contrast to their parent compound, which did not induce any toxicities.^82^ All of the chrysenols assessed in our study, including 1-COH, 2-COH, 3-COH, and 4-COH, were stronger agonists of both Ahr subtypes than unsubstituted chrysene. While it is tempting to explain the observed differences in toxicity of CHR and chrysenols in zebrafish by differences in potential to activate Ahr, this is not straightforward due to the number of possible Ahr-dependent and Ahr-independent mechanisms that could be involved.^74^ However, our findings demonstrated that several chrysenol congeners can act as potent Ahr agonists and may potentially cause toxic effects in Atlantic cod via activation of the Ahr-signaling pathway.

Many of the PACs assessed in this study produced different activation profiles for Ahr1a and Ahr2a. In accordance with our previous findings for β-naphthoflavone and PCB-126,^37^ we found that eight PACs produced the highest efficacies with Ahr1a, while only 2-MOC and 3-MOC produced the highest efficacies with Ahr2a. However, when comparing potencies, Ahr2a displayed higher REP_25_ for several PACs compared to Ahr1a. The observed discrepancies in activation profiles may be ascribed to the relatively low conservation between the two Ahrs. While 31% of the amino acids overall have been conserved between Ahr1a and Ahr2a, 61% have been conserved in the ligand-binding domain (LBD). Interestingly, their TCDD activation profiles also differ, even though the amino acids known to bind and coordinate TCDD are conserved between the two Ahrs,^37^ suggesting that the observed differences must be attributed to features located elsewhere in these proteins. In a similar vein, the observed differences in affinities to TCDD of Ahr variants from different populations of Atlantic tomcod (*Microgadus tomcod*) from Hudson River (NY, USA) could not be ascribed to differences in the LBD but rather to other structural differences that affect the stability of the protein and result in lesser affinity of TCDD.^83^

Molecular mechanisms and physiological effects of PAH exposure have been shown to differ among individual PAH/PAC congeners. Hence, three modes of action have previously been described in teleost species, including Ahr-independent, Ahr-dependent, and Cyp1a metabolism-dependent.^55,74,84,85^ While adverse effects on Atlantic cod have been demonstrated after crude oil and produced water exposure,^86−88^ limited information exists for this species regarding the toxicity mediated by the individual chemical constituents, such as unsubstituted and substituted PACs. However, it was shown in juvenile Atlantic haddock (*Melanogrammus aeglefinus*), which is another Gadiform species, that among 12 injected unsubstituted heavy PAHs, including BAP, benz[*a*]anthracene, dibenz[*a*,*h*,]anthracene, and CHR, produced high levels of DNA adducts in the liver.^89^ Furthermore, it has also been shown that intramuscular injections of NAP, CHR, and their corresponding dihydrodiol metabolites *R*,*R*-1,2-DHN and (1*R*,2*R*)-1,2-dihydrochrysene-1,2-diol result in the formation of PAH-protein adducts in the Atlantic cod plasma proteome and possibly a triggered immune response.^90^ Importantly, it was recently demonstrated that BAP activated the Ahr-signaling pathway in early life stages of Atlantic cod.^38^*Ahr*2*a* expression was induced in the cardiovascular system in both cod embryos and larvae, indicating cardiotoxicity responses in an Ahr-dependent mode of action. This is similar to observations of BAP-mediated activation of Ahr2 in zebrafish embryos, which produced cardiotoxic effects via induction of *cyp*1*a* expression.^91^ Thus, as several PACs are demonstrated in this study to act as Ahr2a agonists with potencies and efficacies in the same range as BAP, including 2-MC, 3-MC, 2-MOC, and 4-MOC, it is not unlikely that exposure to such PAHs during early life stages of cod produces cardiotoxic effects. In contrast to *ahr2*, *ahr1a* transcripts were solely detected in the eye of cod embryos and larvae, and its expression was unaffected by BAP exposure, supporting that Ahr2a is the major subtype involved in mediating PAH-induced responses during early life stages.^38^ Thus, although Ahr1a was shown here to be sensitive toward a wide array of substituted and unsubstituted PACs *in vitro*, it appears that Ahr1a does not have a prominent role in producing adverse effects of PAH exposure during early development. However, it cannot be excluded that activation of Ahr1a may produce adverse effects in Atlantic cod via modulation of yet undescribed Ahr1a-regulated pathways.

In conclusion, we have shown that substituted PAHs, including methylated, mono- and dihydroxylated and methoxylated PACs, are strong agonists of the Atlantic cod Ahrs. The substituted PAHs are also more potent and produce higher efficacies in comparison to their unsubstituted parent compounds. Ahr1a and Ahr2a were mostly activated by the same PACs, but Ahr2a was, in general, the most sensitive receptor, displaying the highest potencies of the compounds. Importantly, our results strongly support that substituted PACs may contribute significantly to the biological effects of PAHs in the environment, and their contribution should be considered when assessing the risk and hazards of PACs. Usually, assessments of the risk and hazard, as well as monitoring of PACs, are based solely on the quantification of the 16 priority PAHs. However, as substituted PACs may contribute significantly to Ahr-mediated toxicity of environmental samples, it becomes apparent that measurement of the 16 priority PAHs is insufficient for predicting PAH-induced toxicity in aquatic environments. Complementing chemical analyses with reporter gene assays as used in this study could significantly aid the risk assessment of environmental samples. Such assays can integrate individual potencies and mixture interactions of compounds that act via a common mode of action. As toxicological data on substituted and heterocyclic PACs is still limited, further studies are necessary to elucidate their mode of action and their joint potencies in mixtures.

## Supporting Information Available

Additional information about dose–response curves, cell viability, statistics, and compound synthesis (PDF)
