# Supplementary material for: Substituted Two- to Five-Ring Polycyclic Aromatic Compounds Are Potent Agonists of Atlantic Cod (Gadus morhua) Aryl Hydrocarbon Receptors Ahr1a and Ahr2a
Source: Environ Sci Technol. 2021 Nov 5;55(22):15123–35. doi: 10.1021/acs.est.1c02946 (PMC8600679; doi:10.1021/acs.est.1c02946)

Supplementary information:

Substituted two- to five-ring polycyclic aromatic compounds are potent agonists of Atlantic cod (*Gadus morhua*) aryl hydrocarbon receptors Ahr1a and Ahr2a

Roger Lille-Langøy[1], Kåre Bredeli Jørgensen[2], Anders Goksøyr[1], Daniela Pampanin[2], Magne O. Sydnes[2] and Odd André Karlsen[1*]

[1]Department of Biological Sciences, University of Bergen, 5020 Bergen, Norway. [2]Department of Chemistry, Bioscience and Environmental Engineering, University of Stavanger, 4036 Stavanger,

[*]Corresponding author, odd.karlsen@uib.no

Supplementary information contents:

Part I – Results and statistics

Supplementary Figure S1 - Dose-response curves for responses induced by known agonists TCDD and FICZ via Atlantic cod Ahr1a and Ahr2a

Supplementary Figure S2 - In vitro activation of Atlantic cod aryl hydrocarbons receptors (Ahrs) by unsubstituted PACs.

Supplementary Figure S3 - In vitro activation of Atlantic cod aryl hydrocarbons receptors (Ahrs) by phenanthrene, naphthalene, and substituted congeners.

Supplementary Figure S4 – COS-7 cell viability by resazurin reduction assay

Supplementary Table S1 - Statistical analysis - Responses (means of EC50 and Emax) via Ahr1a or Ahr2a – TCDD and B[*a*]P

Supplementary Table S2 – Table of *p*-values of statistical analysis - Responses (means of EC50 and Emax) via Ahr1a or Ahr2a – Methylated chrysenes (ANOVA+Dunnet´s test)

Supplementary Table S3 - Table of *p*-values of statistical analysis - Responses (means of EC50 and Emax) via Ahr1a or Ahr2a – Hydroxylated chrysenes (ANOVA+Dunnet´s test)

Supplementary Table S4 - Table of *p*-values of statistical analysis - Responses (means of EC50 and Emax) via Ahr1a or Ahr2a – Methoxylated chrysenes (ANOVA+Dunnet´s test)

Supplementary Table S5 - Table of *p*-values of statistical analysis - Responses (means of EC50 and Emax) via Ahr1a and Ahr2a (Welch´s t-test)

Supplementary Table S6 – Overview of test chemicals**Part II: Synthesis**

General information

Synthesis of 2,3-dimethoxychrysene (S2)

Part I – Results and statistics

Supplementary Figure S1:

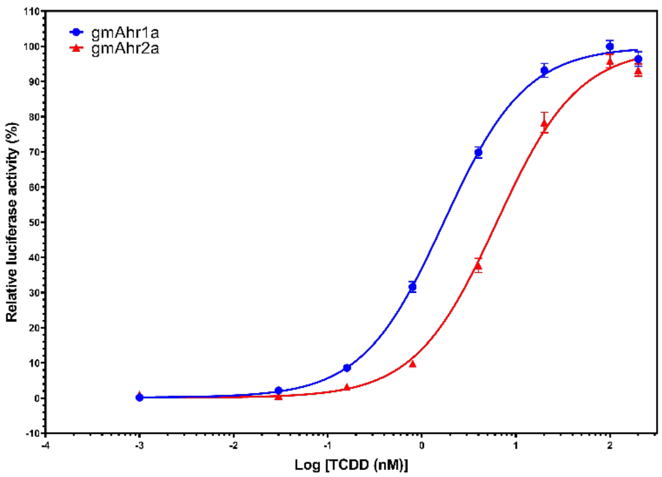

Supplementary Figure S1 - Relative response curves for the luciferase activity response induced by TCDD via of Atlantic cod Ahr1a and Ahr2a. Responses are expressed relative to maximum response induced by TCDD (100%).

Supplementary Figure S2:

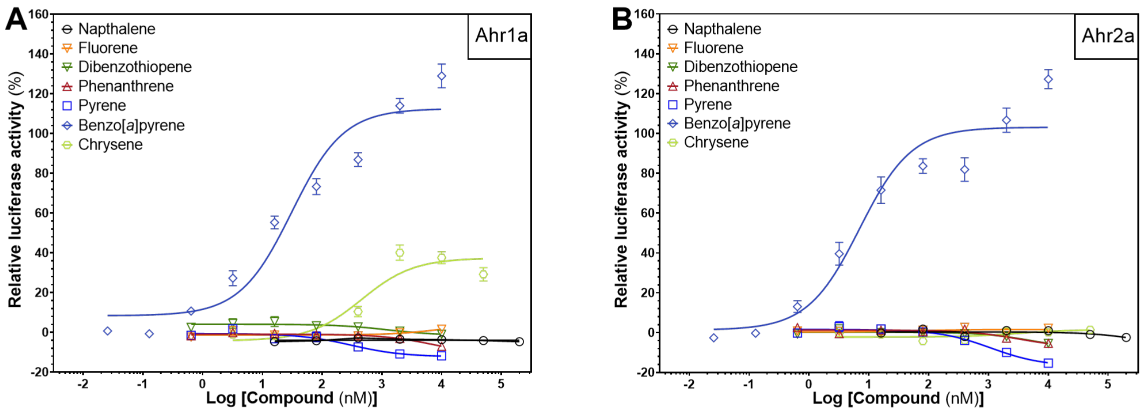

Supplementary Figure S2 – In vitro activation of Atlantic cod aryl hydrocarbons receptors (Ahrs) by unsubstituted PACs. Response curves were recorded with luciferase based Ahr1a (A) and Ahr2a (B) ligand activation assays for seven unsubstituted polycyclic aromatic compounds. Responses are expressed relative to the maximum response induced by 2,3,7,8-tetrachlorodibenzo-p-dioxin (TCDD) (equals 100%).

Supplementary Figure S3:

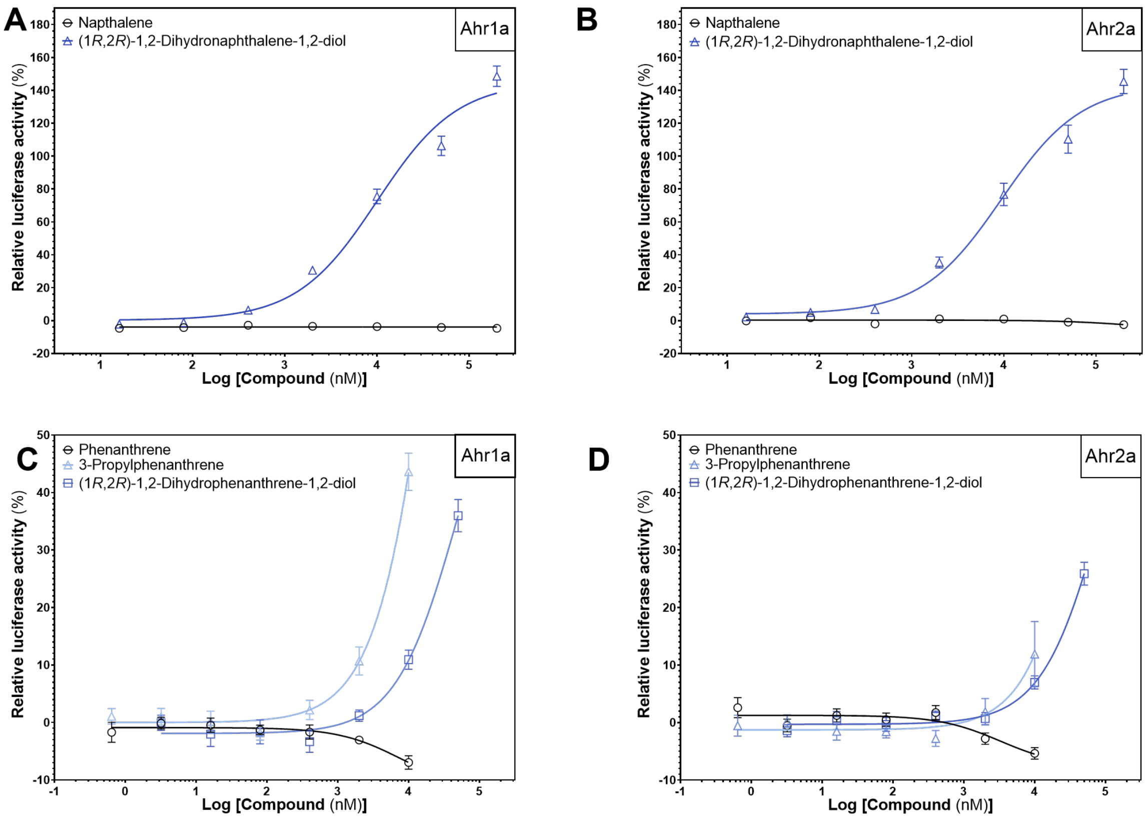

Supplementary Figure S3 – In vitro activation of Atlantic cod aryl hydrocarbons receptors (Ahrs) by phenanthrene, naphthalene, and substituted congeners. Response curves were recorded with luciferase-based Ahr1a and Ahr2a ligand activation assays for naphthalene and (1*R*,2*R*)-1,2-dihydroxynaphthalene-1,2-diol (A&B), as well as phenanthrene, (1*R*,2*R*)-1,2-dihydroxyphenanthrene-1,2-diol, and 3-propylphenanthrene (C&D). Responses are expressed relative to the maximum response induced by TCDD (equals 100%).

Supplementary Figure S4:

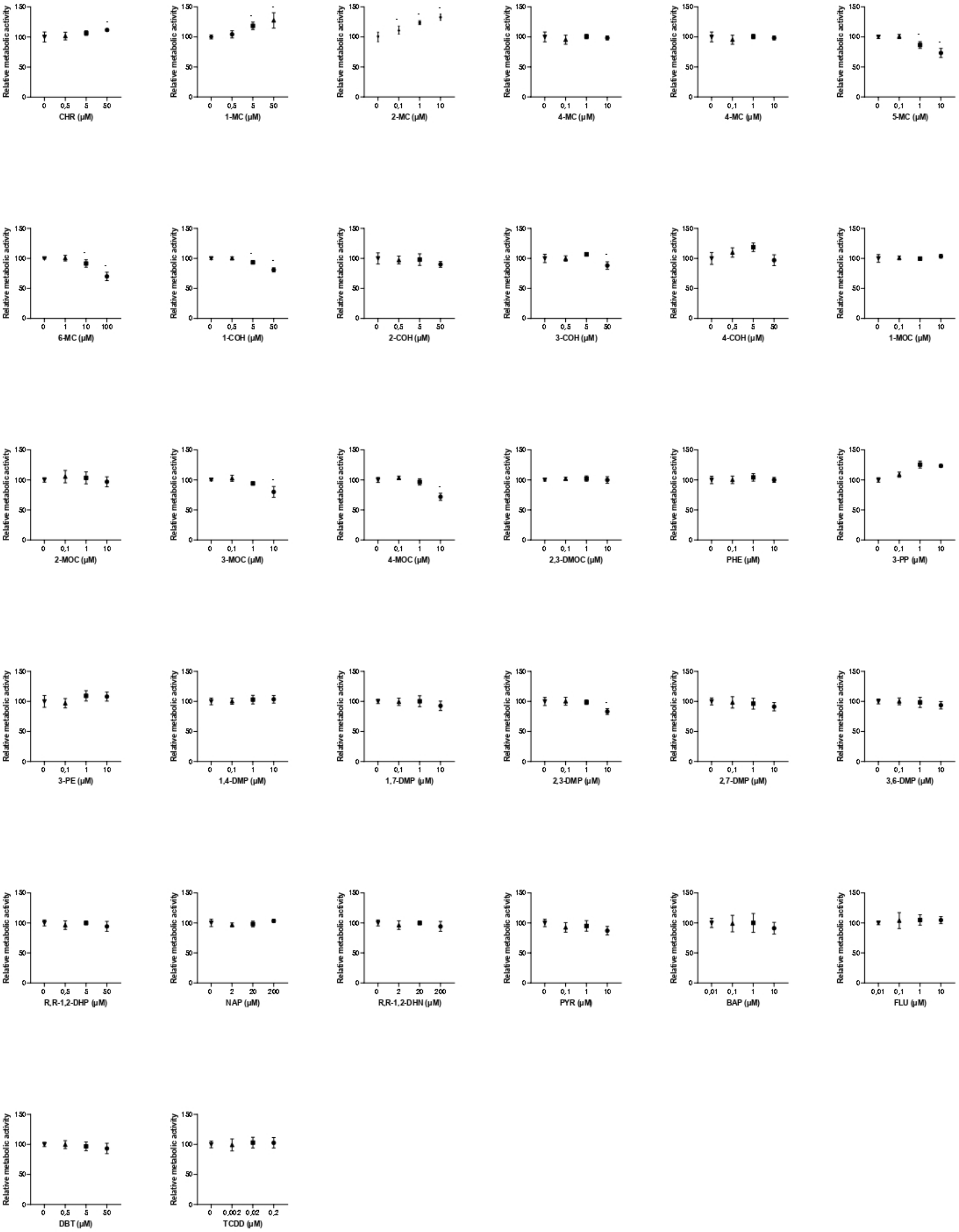

Supplementary Figure S4 - *COS-7 cell viability assay*. Effect on the metabolic activity of COS-7 cells exposed to controls and test compounds as measured by the resazurin reduction assay. Metabolic activities were expressed relative to metabolic activity in cells exposed to 0.5% DMSO/solvent control and statistical significance was calculated by One-way ANOVA corrected by Dunnett´s (p<0.05).

Supplementary Table S1:

Supplementary Table S1 Statistical test of means of E_{max} and EC_{50} for responses induced by TCDD or B[a]P via either Ahr1a or Ahr2a by one-way analysis of variance (ANOVA) and Dunnett's test. The null hypothesis of equal means was rejected when the test showed a 5% probability of the means to be equal (p< 0.05) and have been marked in green in the table.

Emax gmAhr1a	2,3,7,8-Tetrachloro-dibenzodioxin (TCDD)	Benzo-[a]-pyrene
2,3,7,8-Tetrachloro-dibenzodioxin (TCDD)		***0,0211***
Benzo-[a]-pyrene	***0,0211***	

Emax gmAhr2a	2,3,7,8-Tetrachloro-dibenzodioxin (TCDD)	Benzo-[a]-pyrene
2,3,7,8-Tetrachloro-dibenzodioxin (TCDD)		0,5364
Benzo-[a]-pyrene	0,5364	

EC50 gmAhr1a	2,3,7,8-Tetrachloro-dibenzodioxin (TCDD)	Benzo-[a]-pyrene
2,3,7,8-Tetrachloro-dibenzodioxin (TCDD)		***<0,0001***
Benzo-[a]-pyrene	***<0,0001***	

EC50 gmAhr2a	2,3,7,8-Tetrachloro-dibenzodioxin (TCDD)	Benzo-[a]-pyrene
2,3,7,8-Tetrachloro-dibenzodioxin (TCDD)		0,8663
Benzo-[a]-pyrene	0,8663	

Supplementary Table S2:

Supplementary Table S2 Statistical test of means of E_{max} and EC_{50} for responses induced by chrysene and methylated chrysenes via either Ahr1a or Ahr2a by one-way analysis of variance (ANOVA) and Dunnett's test. The null hypothesis of equal means was rejected when the test showed a 5% probability of the means to be equal (*p*< 0.05) and have been marked in green in the table.

Emax gmAhr1a	2,3,7,8-Tetrachloro-dibenzodioxin (TCDD)	Chrysene	1-methylchrysene	2-methylchrysene	3-methylchrysene	4-methylchrysene	5-methylchrysene	6-methylchrysene
2,3,7,8-Tetrachloro-dibenzodioxin (TCDD)		<0,0001	<0,0001	<0,0001	0,1002	0,0178	0,2818	0,2434
Chrysene	<0,0001		0,9666	<0,0001	<0,0001	<0,0001	<0,0001	<0,0001
1-methylchrysene	<0,0001	0,9666		<0,0001	<0,0001	<0,0001	<0,0001	<0,0001
2-methylchrysene	<0,0001	<0,0001	<0,0001		0,072	0,1849	<0,0001	<0,0001
3-methylchrysene	0,1002	<0,0001	<0,0001	0,072		0,9999	0,0016	0,0009
4-methylchrysene	0,0178	<0,0001	<0,0001	0,1849	0,9999		0,0002	0,0001
5-methylchrysene	0,2818	<0,0001	<0,0001	<0,0001	0,0016	0,0002		>0,9999
6-methylchrysene	0,2434	<0,0001	<0,0001	<0,0001	0,0009	0,0001	>0,9999	

Emax gmAhr2a	2,3,7,8-Tetrachloro-dibenzodioxin (TCDD)	Chrysene	1-methylchrysene	2-methylchrysene	3-methylchrysene	4-methylchrysene	5-methylchrysene	6-methylchrysene
2,3,7,8-Tetrachloro-dibenzodioxin (TCDD)		<0,0001	<0,0001	<0,0001	0,9988	<0,0001	<0,0001	0,1783
Chrysene	<0,0001		<0,0001	<0,0001	<0,0001	<0,0001	<0,0001	<0,0001
1-methylchrysene	<0,0001	<0,0001		<0,0001	<0,0001	0,1519	<0,0001	<0,0001
2-methylchrysene	<0,0001	<0,0001	<0,0001		<0,0001	<0,0001	<0,0001	<0,0001
3-methylchrysene	0,9988	<0,0001	<0,0001	<0,0001		<0,0001	<0,0001	0,1844
4-methylchrysene	<0,0001	<0,0001	0,1519	<0,0001	<0,0001		0,1865	<0,0001
5-methylchrysene	<0,0001	<0,0001	<0,0001	<0,0001	<0,0001	0,1865		0,0005
6-methylchrysene	0,1783	<0,0001	<0,0001	<0,0001	0,1844	<0,0001	0,0005	

EC50 gmAhr1a	2,3,7,8-Tetrachloro-dibenzodioxin (TCDD)	Chrysene	1-methylchrysene	2-methylchrysene	3-methylchrysene	4-methylchrysene	5-methylchrysene	6-methylchrysene
2,3,7,8-Tetrachloro-dibenzodioxin (TCDD)		<0,0001	<0,0001	<0,0001	<0,0001	<0,0001	<0,0001	<0,0001
Chrysene	<0,0001		0,0037	<0,0001	<0,0001	<0,0001	0,995	<0,0001
1-methylchrysene	<0,0001	0,0037		<0,0001	0,4342	0,8474	0,0004	<0,0001
2-methylchrysene	<0,0001	<0,0001	<0,0001		<0,0001	<0,0001	<0,0001	<0,0001
3-methylchrysene	<0,0001	<0,0001	0,4342	<0,0001		0,9981	<0,0001	<0,0001
4-methylchrysene	<0,0001	<0,0001	0,8474	<0,0001	0,9981		<0,0001	<0,0001
5-methylchrysene	<0,0001	0,995	0,0004	<0,0001	<0,0001	<0,0001		<0,0001
6-methylchrysene	<0,0001	<0,0001	<0,0001	<0,0001	<0,0001	<0,0001	<0,0001	

EC50 gmAhr2a	2,3,7,8-Tetrachloro-dibenzodioxin (TCDD)	Chrysene	1-methylchrysene	2-methylchrysene	3-methylchrysene	4-methylchrysene	5-methylchrysene	6-methylchrysene
2,3,7,8-Tetrachloro-dibenzodioxin (TCDD)		<0,0001	<0,0001	0,1446	0,0481	0,0006	0,0003	0,0045
Chrysene	<0,0001		0,0159	<0,0001	<0,0001	0,0048	0,0444	0,0009
1-methylchrysene	<0,0001	0,0159		0,7608	0,8938	>0,9999	>0,9999	0,9955
2-methylchrysene	0,1446	<0,0001	0,7608		>0,9999	0,9165	0,7404	0,9904
3-methylchrysene	0,0481	<0,0001	0,8938	>0,9999		0,9777	0,874	0,9992
4-methylchrysene	0,0006	0,0048	>0,9999	0,9165	0,9777		0,9999	>0,9999
5-methylchrysene	0,0003	0,0444	>0,9999	0,7404	0,874	0,9999		0,9915
6-methylchrysene	0,0045	0,0009	0,9955	0,9904	0,9992	>0,9999	0,9915	

Supplementary Table S3:

Supplementary Table S3 Statistical test of means of E_{max} and EC_{50} for responses induced by chrysene and hydroxylated chrysenes via either Ahr1a or Ahr2a by one-way analysis of variance (ANOVA) and Dunnett's test. The null hypothesis of equal means was rejected when the test showed a 5% probability of the means to be equal ($p < 0.05$) and have been marked in green in the table.

Emax gmAhr1a	2,3,7,8-Tetrachloro-dibenzodioxin (TCDD)	Chrysene	Chrysene-1-ol	Chrysene-2-ol	Chrysene-3-ol	Chrysene-4-ol
2,3,7,8-Tetrachloro-dibenzodioxin (TCDD)		***<0,0001***	***<0,0001***	0,0784	***0,0006***	0,1317
Chrysene	***<0,0001***		***<0,0001***	***<0,0001***	***<0,0001***	***<0,0001***
Chrysene-1-ol	***<0,0001***	***<0,0001***		***<0,0001***	***<0,0001***	***<0,0001***
Chrysene-2-ol	0,0784	***<0,0001***	***<0,0001***		0,8759	>0,9999
Chrysene-3-ol	***0,0006***	***<0,0001***	***<0,0001***	0,8759		0,7964
Chrysene-4-ol	0,1317	***<0,0001***	***<0,0001***	>0,9999	0,7964	

Emax gmAhr2a	2,3,7,8-Tetrachloro-dibenzodioxin (TCDD)	Chrysene	Chrysene-1-ol	Chrysene-2-ol	Chrysene-3-ol	Chrysene-4-ol
2,3,7,8-Tetrachloro-dibenzodioxin (TCDD)		***<0,0001***	***<0,0001***	***<0,0001***	***<0,0001***	>0,9999
Chrysene	***<0,0001***		***<0,0001***	***<0,0001***	***<0,0001***	***<0,0001***
Chrysene-1-ol	***<0,0001***	***<0,0001***		***<0,0001***	0,8904	***0,0004***
Chrysene-2-ol	***<0,0001***	***<0,0001***	***<0,0001***		***0,0007***	***<0,0001***
Chrysene-3-ol	***<0,0001***	***<0,0001***	0,8904	***0,0007***		***<0,0001***
Chrysene-4-ol	>0,9999	***<0,0001***	***0,0004***	***<0,0001***	***<0,0001***	

EC50 gmAhr1a	2,3,7,8-Tetrachloro-dibenzodioxin (TCDD)	Chrysene	Chrysene-1-ol	Chrysene-2-ol	Chrysene-3-ol	Chrysene-4-ol
2,3,7,8-Tetrachloro-dibenzodioxin (TCDD)		***<0,0001***	***<0,0001***	***<0,0001***	***<0,0001***	***<0,0001***
Chrysene	***<0,0001***		>0,9999	0,604	0,7568	***0,0005***
Chrysene-1-ol	***<0,0001***	>0,9999		0,6414	0,7818	***0,0014***
Chrysene-2-ol	***<0,0001***	0,604	0,6414		>0,9999	***<0,0001***
Chrysene-3-ol	***<0,0001***	0,7568	0,7818	>0,9999		***<0,0001***
Chrysene-4-ol	***<0,0001***	***0,0005***	***0,0014***	***<0,0001***	***<0,0001***	

EC50 gmAhr2a	2,3,7,8-Tetrachloro-dibenzodioxin (TCDD)	Chrysene	Chrysene-1-ol	Chrysene-2-ol	Chrysene-3-ol	Chrysene-4-ol
2,3,7,8-Tetrachloro-dibenzodioxin (TCDD)		***<0,0001***	***<0,0001***	***<0,0001***	***0,0003***	***<0,0001***
Chrysene	***<0,0001***		0,5999	0,2997	0,1628	0,8845
Chrysene-1-ol	***<0,0001***	0,5999		0,9976	0,9762	0,111
Chrysene-2-ol	***<0,0001***	0,2997	0,9976		0,9997	***0,0325***
Chrysene-3-ol	***0,0003***	0,1628	0,9762	0,9997		***0,0131***
Chrysene-4-ol	***<0,0001***	0,8845	0,111	***0,0325***	***0,0131***	

Supplementary Table S4:

Supplementary Table S 4 Statistical test of means of E_{max} and EC_{50} for responses induced by chrysene and methoxylated chrysenes via either Ahr1a or Ahr2a by one-way analysis of variance (ANOVA) and Dunnett's test. The null hypothesis of equal means was rejected when the test showed a 5% probability of the means to be equal (p< 0.05) and have been marked in green in the table.

Emax gmAhr1a	2,3,7,8-Tetrachloro-dibenzodioxin (TCDD)	Chrysene	1-methoxychrysene	2-methoxychrysene	3-methoxychrysene	4-methoxychrysene	2,3-dimethoxychrysene
2,3,7,8-Tetrachloro-dibenzodioxin (TCDD)		***<0,0001***	0,9936	0,9329	***<0,0001***	***0,0001***	***<0,0001***
Chrysene	***<0,0001***		***<0,0001***	***<0,0001***	***<0,0001***	***<0,0001***	***<0,0001***
1-methoxychrysene	0,9936	***<0,0001***		>0,9999	***<0,0001***	***0,0489***	***0,0003***
2-methoxychrysene	0,9329	***<0,0001***	>0,9999		***<0,0001***	0,1112	***0,0012***
3-methoxychrysene	***<0,0001***	***<0,0001***	***<0,0001***	***<0,0001***		***0,0036***	0,2121
4-methoxychrysene	***0,0001***	***<0,0001***	***0,0489***	0,1112	***0,0036***		0,8137
2,3-dimethoxychrysene	***<0,0001***	***<0,0001***	***0,0003***	***0,0012***	0,2121	0,8137	

Emax gmAhr2a	2,3,7,8-Tetrachloro-dibenzodioxin (TCDD)	Chrysene	1-methoxychrysene	2-methoxychrysene	3-methoxychrysene	4-methoxychrysene	2,3-dimethoxychrysene
2,3,7,8-Tetrachloro-dibenzodioxin (TCDD)		***<0,0001***	***<0,0001***	***0,0095***	***0,0004***	0,8407	***<0,0001***
Chrysene	***<0,0001***		***<0,0001***	***<0,0001***	***<0,0001***	***<0,0001***	***<0,0001***
1-methoxychrysene	***<0,0001***	***<0,0001***		***<0,0001***	0,1471	***<0,0001***	0,4079
2-methoxychrysene	***0,0095***	***<0,0001***	***<0,0001***		***<0,0001***	***0,0047***	***<0,0001***
3-methoxychrysene	***0,0004***	***<0,0001***	0,1471	***<0,0001***		0,2575	***0,0001***
4-methoxychrysene	0,8407	***<0,0001***	***<0,0001***	***0,0047***	0,2575		***<0,0001***
2,3-dimethoxychrysene	***<0,0001***	***<0,0001***	0,4079	***<0,0001***	***0,0001***	***<0,0001***	

EC50 gmAhr1a	2,3,7,8-Tetrachloro-dibenzodioxin (TCDD)	Chrysene	1-methoxychrysene	2-methoxychrysene	3-methoxychrysene	4-methoxychrysene	2,3-dimethoxychrysene
2,3,7,8-Tetrachloro-dibenzodioxin (TCDD)		***<0,0001***	***<0,0001***	***<0,0001***	***<0,0001***	***<0,0001***	***<0,0001***
Chrysene	***<0,0001***		***<0,0001***	***<0,0001***	***0,0177***	***<0,0001***	***0,0226***
1-methoxychrysene	***<0,0001***	***<0,0001***		***<0,0001***	0,3535	0,0852	0,3144
2-methoxychrysene	***<0,0001***	***<0,0001***	***<0,0001***		***<0,0001***	***0,0016***	***<0,0001***
3-methoxychrysene	***<0,0001***	***0,0177***	0,3535	***<0,0001***		***<0,0001***	>0,9999
4-methoxychrysene	***<0,0001***	***<0,0001***	0,0852	***0,0016***	***<0,0001***		***<0,0001***
2,3-dimethoxychrysene	***<0,0001***	***0,0226***	0,3144	***<0,0001***	>0,9999	***<0,0001***	

EC50 gmAhr2a	2,3,7,8-Tetrachloro-dibenzodioxin (TCDD)	Chrysene	1-methoxychrysene	2-methoxychrysene	3-methoxychrysene	4-methoxychrysene	2,3-dimethoxychrysene
2,3,7,8-Tetrachloro-dibenzodioxin (TCDD)		***<0,0001***	***0,0186***	0,8997	0,0789	0,6709	***0,0009***
Chrysene	***<0,0001***		0,111	***0,0002***	***0,035***	***0,001***	0,4424
1-methoxychrysene	***0,0186***	0,111		0,6802	0,9998	0,8645	0,9961
2-methoxychrysene	0,8997	***0,0002***	0,6802		0,8833	0,9999	0,2823
3-methoxychrysene	0,0789	***0,035***	0,9998	0,8833		0,973	0,9544
4-methoxychrysene	0,6709	***0,001***	0,8645	0,9999	0,973		0,4839
2,3-dimethoxychrysene	***0,0009***	0,4424	0,9961	0,2823	0,9544	0,4839	

Supplementary Table S5:

Supplementary Table S5 – Comparison of efficacy and potency of in vitro responses of selected polycyclic aromatic compounds via aryl hydrocarbon receptor Ahr1a or Ahr2a. Welch´s t-test was used to test the hypothesis that means of recorded E_{max} and EC_{50}s were equal. The hypothesis rejection cut-off was set to 5% probability of the means to be equal ($p<0.05$), and rejected test were marked in green in the table. For Ahr1a or Ahr2a responses that were significantly different, the higher efficacy (E_{max}) and the highest potency (lowest EC_{50}) were marked in red. Three test compounds with agonistic potential, 3-propylphenanthrene, (1*R*,2*R*)-1,2-dihydrophenanthrene-1.2-diol and (1*R*,2*R*)-1,2-dihydronaphthalene-1,2-diol, were omitted from this overview because their dose-response curves did not reach a plateau.

		Chrysene	1-methylchrysene	2-methylchrysene	3-methylchrysene	4-methylchrysene	5-methylchrysene	6-methylchrysene	1-hydroxychrysene	2-hydroxychrysene	3-hydroxychrysene	4-hydroxychrysene	1-methoxychrysene	2-methoxychrysene	3-methoxychrysene	4-methoxychrysene	2,3-dimethoxychrysene	Benzo-[a]-pyrene
Efficacy (Emax, %)	Ahr1a	40,8	45,8	124,6	110,5	112,5	90,1	90,6	115,9	91,1	86,5	91,7	97,3	95,9	66,6	83,8	77,5	106,7
	Ahr2a	3,6	43,6	126,9	101,7	55,9	68,4	89,8	72,8	40,8	66,1	98,8	66,4	114,5	80,8	93,7	55,0	102,7
	Welch´s t-test p-value	***<0,0001***	0,6217	0,7552	0,0693	***<0,0001***	***0,0004***	0,8143	***<0,0001***	***<0,0001***	***<0,0001***	0,4826	***<0,0001***	***0,0183***	***0,0147***	0,066	***<0,0001***	0,2052
Potency (EC50, nM)	Ahr1a	481,2	175,0	22,0	100,9	120,0	595,9	3536	484,5	324,4	344,6	1344	102,3	13,0	195,0	43,8	199,0	25,8
	Ahr2a	3442	171,7	41,8	54,0	127,7	193,2	88,8	632,6	380,0	274,5	10975	160,2	20,1	98,6	30,9	359,1	6,8
	Welch´s t-test p-value	0,2994	0,9711	***0,0252***	***0,0055***	0,8666	***0,0031***	***<0,0001***	0,3722	0,7227	0,4958	***<0,0001***	0,1399	0,2231	0,0616	0,238	0,1075	***<0,0001***

Supplementary Table S6:

Supplementary Table S6 – Comparison of the efficacy and potency of test compound and TCDD in in vitro responses mediated by aryl hydrocarbon receptor Ahr1a or Ahr2a. Welch´s t-test was used to test the hypothesis that means of recorded E_{max} and EC_{50}s were equal. The hypothesis rejection cut-off was set to 5% probability of the means to be equal ($p<0.05$). Test compound with efficacy or potency that were significantly different from those TCDD, were marked in bold italic. Green signifies that the test compound had lower E_{max} or EC_{50} than TCDD, while red signifies that the test compound had higher E_{max} or EC_{50} than TCDD.

**Efficacy of test compound vs TCDD**	Chrysene	1-methylchrysene	2-methylchrysene	3-methylchrysene	4-methylchrysene	5-methylchrysene	6-methylchrysene	Chrysene-1-ol	Chrysene-2-ol	Chrysene-3-ol	Chrysene-4-ol	1-methoxychrysene	2-methoxychrysene	3-methoxychrysene	4-methoxychrysene	2,3-dimethoxychrysene	Benzo-[a]-pyrene
gmAhr1a	***<0,0001***	***<0,0001***	***<0,0001***	0,1002	***0,0178***	0,2818	0,2434	***<0,0001***	0,0784	***0,0006***	0,1317	0,9936	0,9329	***<0,0001***	***0,0001***	***<0,0001***	***0,0211***
gmAhr2a	***<0,0001***	***<0,0001***	***<0,0001***	0,9988	***<0,0001***	***<0,0001***	0,1783	***<0,0001***	***<0,0001***	***<0,0001***	>0,9999	***<0,0001***	***0,0095***	***0,0004***	0,8407	***<0,0001***	0,5364

**Potency of test compound vs TCDD**	Chrysene	1-methylchrysene	2-methylchrysene	3-methylchrysene	4-methylchrysene	5-methylchrysene	6-methylchrysene	Chrysene-1-ol	Chrysene-2-ol	Chrysene-3-ol	Chrysene-4-ol	1-methoxychrysene	2-methoxychrysene	3-methoxychrysene	4-methoxychrysene	2,3-dimethoxychrysene	Benzo-[a]-pyrene
gmAhr1a	***<0,0001***	***<0,0001***	***<0,0001***	***<0,0001***	***<0,0001***	***<0,0001***	***<0,0001***	***<0,0001***	***<0,0001***	***<0,0001***	***<0,0001***	***<0,0001***	***<0,0001***	***<0,0001***	***<0,0001***	***<0,0001***	***<0,0001***
gmAhr2a	***<0,0001***	***<0,0001***	0,1446	***0,0481***	***0,0006***	***0,0003***	***0,0045***	***<0,0001***	***<0,0001***	***0,0003***	***<0,0001***	***0,0186***	0,8997	0,0789	0,6709	***0,0009***	0,8663

Part II: Synthesis

General information

The photochemical reactions were performed in a Photochemical Reactors Ltd. 400 W medium pressure Mercury-lamp in a 2 L quartz immersion well reactor fitted with a no. 3408 glass filter sleeve. Flash chromatography were performed on silica gel Silice 60A C.C. 40-43 mm from SDS with eluents as described in the procedures. Melting points were obtained in sealed capillary tubes on a Stuart Scientific melting point apparatus SMP3. NMR-spectra were measured on a Varian Mercury 300MHz instrument with tetramethylsilane (0 ppm) as internal reference. HRMS was obtained by ESI+ ionization on a TOF-instrument.

Synthesis of 2,3-dimethoxychrysene (S2):

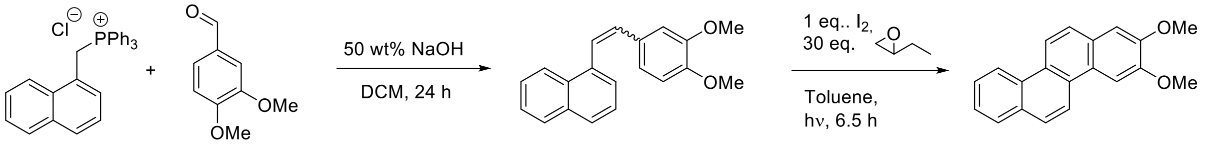

S1 S2

3,4-dimethoxybenzaldehyde (2.55 g, 15.4 mmol) and (naphthalen-1-ylmethyl)triphenylphosphoniumchloride (8.02 g, 18.3 mmol) were dissolved in DCM(120 mL) and vigorously stirred with 50 wt% aqueous NaOH (12 mL) under nitrogen for 24 h. The reaction mixture was added water (400 mL) and extracted. The water phase was extracted with DCM (150 mL). The combined DCM-phases were dried with anhydrous $MgSO_4$ and concentrated under reduced pressure. The remains were purified by flash chromatography (Heptane: ethyl acetate 2:1) to yield 4.43 g (99%) of cis/trans-1-(3,4-dimethoxystyryl)naphthalene (**S1**) an amorphous solid.

Stilbene **S1** (4.43 g, 15.3 mmol), I2 (4.04 g, mmol) and 1,2-epoxybutane (50 mL) was dissolved in degassed toluene (1.2 L) in the photochemical reactor. This reaction mixture was irradiated under nitrogen atmosphere for 6.5 h, until the colour of I_2 had disappeared. The reaction mixture was reduced to 400 mL under reduced pressure, washed with 10% aqueous $Na_2S_2O_3$ (200 mL) followed by brine (100 mL), dried over anhydrous $MgSO_4$ and concentrated under reduced pressure. The remains were purified by flash chromatography (Heptane: ethyl acetate 15:1) to yield 4.11 g (93%) of a 3.2:1 mixture (measured by integration of ^{1}H NMR) of 2,3-

dimethoxychrysene and 3,4-dimethoxychrysene. These isomers were not separable by flash chromatography, but the major product could be isolated from the mixture upon crystallization from acetone to give 0.3079 g (10%) of pure 2,3-dimethoxychrysene (S2) as a colourless powder.

Melting point: 212-213.5 °C (acetone).

IR(KBr): 3000, 2964, 2953, 2842, 1619, 1517(m), 1495(m), 1466, 1456, 1389, 1273(m), 1220(m), 1199, 1159(m), 1039, 862, 811, 758 cm^{-1}.

^{1}H-NMR(300 MHz, Acetone-d6): 4.01(s, 3H), 4.12(s, 3H), 7.51(s, 1H), 7.63(ddd, J=8.0, 7.9, 1.1 Hz, 1H), 7,74(ddd, J=8.3, 7.0, 1.4 Hz, 1H), 7.98-8.05(m, 3H), 8.27(s, 1H), 8.71(d, J=9.0 Hz, 1H), 8.78(d, J= 9.2 Hz), 8.88(d, J= 8.4 Hz, 1H) ppm.

^{13}C-NMR(75 MHz, Acetone-d6):56.0, 56.2, 104.5, 108.9, 120.1, 122.5, 123.9, 126.5(C), 126.9, 127.3, 127.5, 127.6, 128.0(C), 128.5(C), 128.8(C), 129.3, 131.7(C), 132.7(C), 151.0(C), 151.3(C) ppm.

HRMS(ESI^{+}-TOF): m/z $[M+H]^{+}$ for $C_{20}H_{17}O_{2}$: Calcd 289.12285, found 289.12304.

^{1}H-NMR of **S2**:

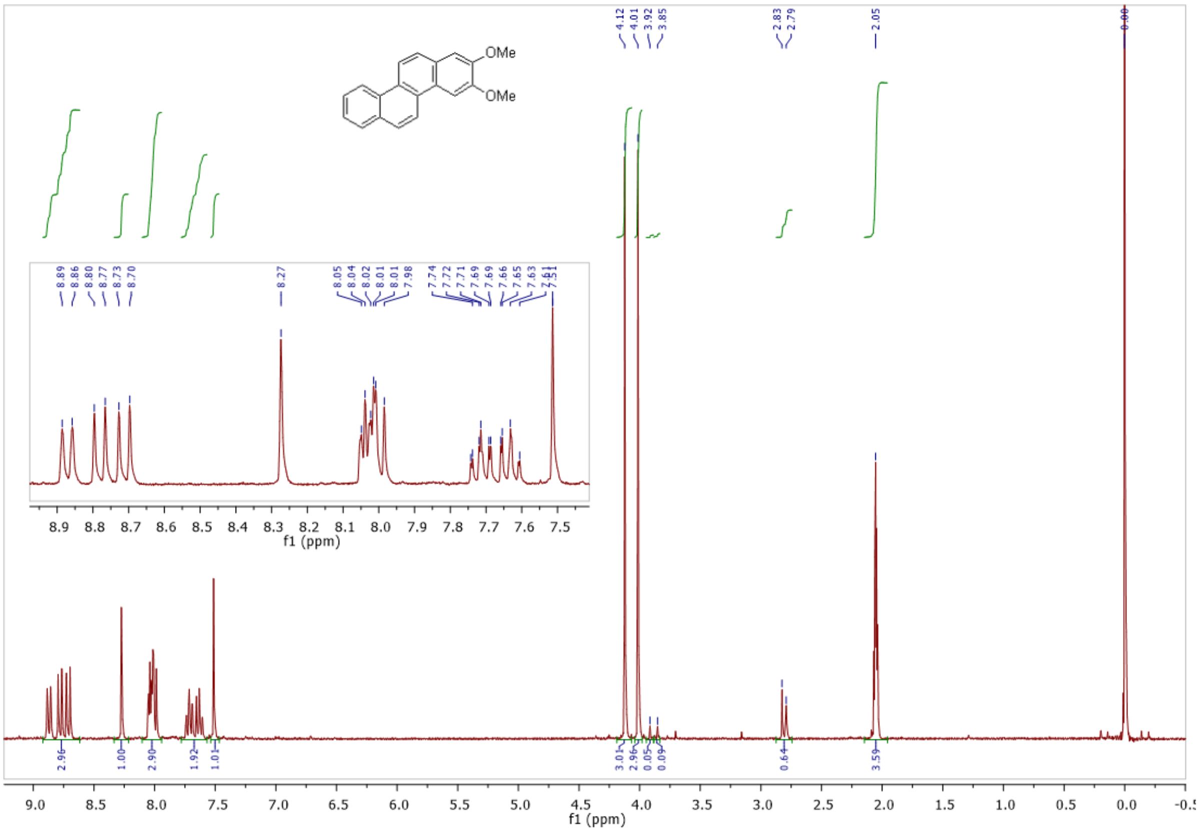

^{13}C-NMR of **S2**:

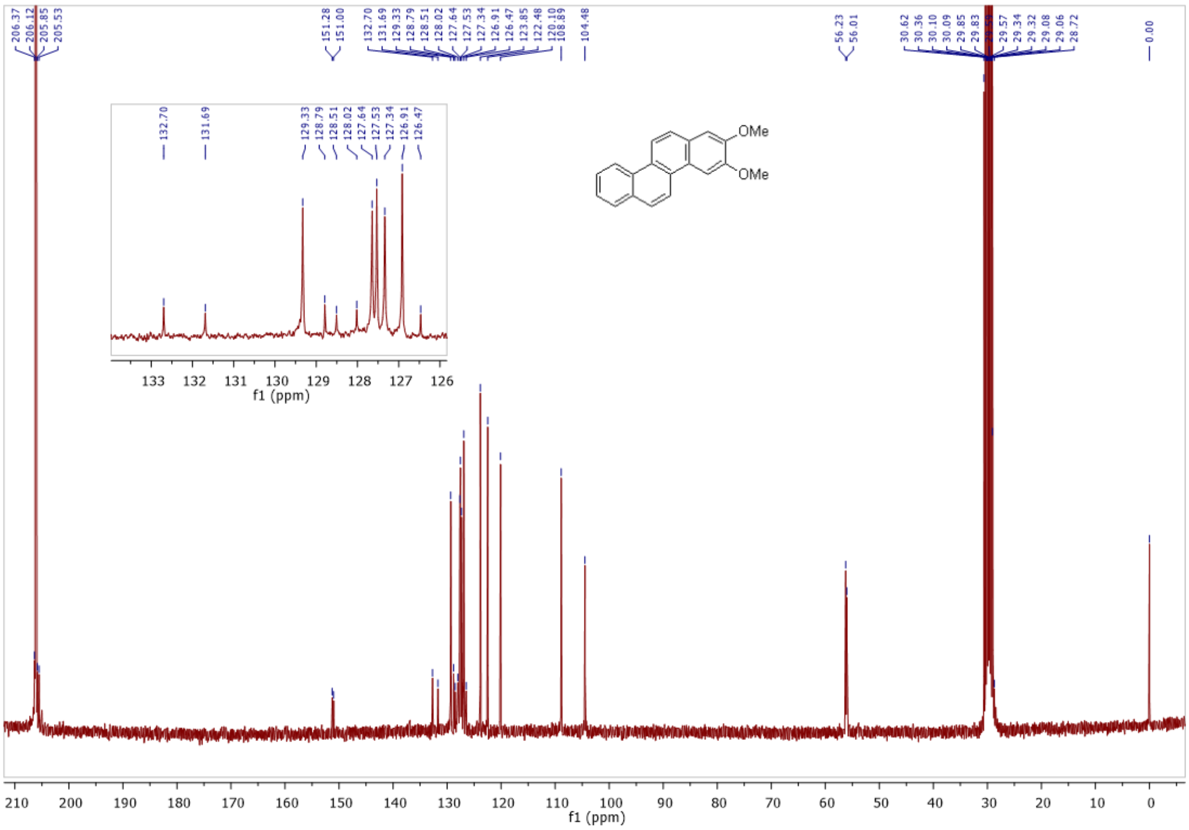

Supplement: Supplementary file 1 — es1c02946_si_001.pdf [file es1c02946_si_001.pdf]
